# Overlap between angina without obstructive coronary artery disease and left ventricular diastolic dysfunction with preserved ejection fraction

**DOI:** 10.1371/journal.pone.0216240

**Published:** 2019-05-23

**Authors:** Marie Mide Michelsen, Adam Pena, Naja D. Mygind, Nis Høst, Ida Gustafsson, Peter Riis Hansen, Henrik Steen Hansen, Jens Kastrup, Eva Prescott

**Affiliations:** 1 Department of Cardiology, Bispebjerg Hospital, University of Copenhagen, Copenhagen, Denmark; 2 Department of Cardiology, Herlev-Gentofte Hospital, University of Copenhagen, Copenhagen, Denmark; 3 Department of Cardiology, Rigshospitalet, University of Copenhagen, Copenhagen, Denmark; 4 Department of Cardiology, Hvidovre Hospital, University of Copenhagen, Copenhagen, Denmark; 5 Department of Cardiology, Odense University Hospital, Odense, Denmark; University of Bologna, ITALY

## Abstract

**Background:**

A link between angina with no obstructive coronary artery disease (CAD) and heart failure with preserved left ventricular ejection fraction has been proposed, but evidence in support of this is lacking. In a cross-sectional study, we investigated whether left ventricular diastolic function in women with angina pectoris and no obstructive CAD differed from a reference population.

**Methods:**

We included 956 women with angina and <50% coronary artery stenosis at invasive coronary angiography. Women with cardiovascular risk factors, but no history of chest pain or cardiac disease served as controls (n = 214). Left ventricular diastolic function was assessed by transthoracic echocardiography.

**Results:**

The women with angina were slightly older, had higher body mass index, higher heart rate, and more had diabetes compared with controls while systolic blood pressure was lower. In age-adjusted analyses, angina patients had significantly lower E/A (Estimated difference -0.13, 95% CI: -0.17; -0.08), higher left ventricular mass index (5.73 g/m^2^, 95% CI: 3.71; 7.75), left atrial volume index (2.34 ml/m^2^, 95% CI: 1.23; 3.45) and E/e’ (0.68, 95% CI: 0.30; 1.05) and a larger proportion had higher estimated left ventricular filling pressure (17% versus 6%, p = 0.001). No between group differences were seen for e’ or deceleration time. After adjustment for known cardiovascular risk factors, between group differences for echocardiographic parameters remained statistically significant.

**Conclusions:**

Patients with angina and no obstructive CAD had a more impaired left ventricular diastolic function compared with an asymptomatic reference population. This suggests some common pathophysiological pathway between the two syndromes.

## Introduction

Women with chest pain suggestive of angina pectoris often undergo multiple examinations for cardiovascular (CV) disease and yet end up without a diagnosis but continued symptoms. Up to 65% of women referred to coronary angiography have insignificant coronary artery stenosis, as compared with 32% of men [1]. Nevertheless, these women have an increased risk of major adverse CV events [2, 3]. Despite increasing interest in the combined presentation of angina and no obstructive coronary artery disease (CAD), the underlying pathophysiologic mechanisms remain unclear.

A large proportion of these women have non-endothelial or endothelial dependent coronary microvascular dysfunction (CMD) [4, 5], which has been suggested to contribute to myocardial abnormalities such as left ventricular (LV) hypertrophy and fibrosis leading to LV diastolic dysfunction. Small echocardiographic studies in patients with microvascular angina (n = 7 and n = 45) reported that LV diastolic function was impaired compared with asymptomatic controls [6, 7]. A more recent cardiac magnetic resonance (CMR) study also showed that women with signs and symptoms of ischemia in the absence of obstructive CAD (n = 20) had abnormalities in diastolic function compared with age-matched controls (n = 15) [8]. However, larger studies assessing LV diastolic function in patients with angina and no obstructive CAD are lacking.

In view of this limited evidence, we investigated whether LV diastolic function assessed by comprehensive transthoracic echocardiography (TTE) differed between women with angina without obstructive CAD and an asymptomatic reference population with CV risk factors.

## Methods

### Population

**Fig 1:**
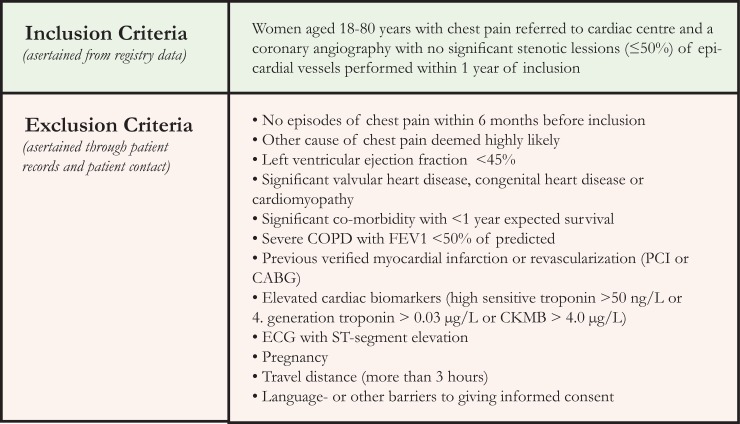
Inclusion and exclusion criteria in the iPOWER study.

Women (18–80 years) suspected of angina pectoris referred for a diagnostic invasive coronary angiography (CAG) showing no significant stenosis (<50%) were systematically included in this substudy of the iPOWER (*i*m*p*r*o*ving diagnosis and treatment of *w*omen with angina p*e*ctoris and microvascula*r* disease) study between March 2012 and September 2014. Recruitment to the prospective iPOWER cohort was consecutive from all invasive centers in Eastern Denmark covering approximately 3 million inhabitants. In- and exclusion criteria are displayed in Fig 1. Further details can be found in previous publications [4, 9]. Participants with no TTE were excluded from this substudy.

For the reference group, we included women aged 40–80 years from a background population with CV risk factors but no prior history of chest pain, dyspnea or cardiac disease, who had participated in the community-based Copenhagen City Heart Study in 2012–14 [10].

### Basic examination

For the women with angina, information regarding symptoms of angina was obtained and classified as typical angina pectoris, atypical angina pectoris and non-cardiac chest pain [11]. Basic assessment in both the women with angina and the controls included clinical and demographic data. CV risk factors including age, body mass index (BMI), diabetes, hypertension, smoking, and family history of CV disease was acquired from interviews undertaken by trained health professionals and patient charts. Blood pressure was obtained at rest. Heart rates were extracted from the continuous electrocardiogram (ECG) registered during the TTE at resting conditions. Patients with angina had paused their beta-blocker and anti-hypertensive medicine for 24 hours prior to the examination.

### Echocardiographic examination

Both angina patients and controls underwent a standard resting TTE using GE Healthcare Vivid E9 CV ultrasound system (GE Healthcare, Horten, Norway) with a 1.3–4.0 MHz transducer (GE Vivid 5S probe). Images were stored for off-line analysis (GE EchoPAC v.112, Norway). A small team of investigators performed image acquisition. Two echocardiographers performed all analyses blinded to baseline characteristics. We have reported good inter-observer reproducibility for echocardiographic measurements between the two experts performing the analyses for this current study [9].

Measurements of LV internal dimensions, LV mass index (LVMI) and left atrium volume index (LAVI) determined by the ‘Volume Method of Discs’ were performed and calculated according to European and American recommendations [12–14]. Relative wall thickness was assessed as the posterior and the septum wall thickness in diastole divided by the left ventricular internal diameter in diastole: (IVSDd + LVPWd)/LVIDd.

Echocardiographic parameters of diastolic function including the early (E) and late (A) mitral inflow velocities, tissue Doppler early and late diastolic velocities in the lateral mitral annulus (e’ and a’) and the E/A and e’/a’-ratios were used as surrogates of myocardial relaxation and LV compliance, the deceleration time as a surrogate of early LV stiffness and, E/e’ as a surrogate estimate of LV filling pressures [14]. All measures were averaged over 3 heart cycles. In case of fusion of the E and A or e’ and a’ waves (as for patients with atrial fibrillation at examination), measurements were registered as missing.

LV filling pressure was categorized as normal or high by using a modified algorithm from the recommendations of the American and European Societies of Echocardiography concerning patients with preserved LV ejection fraction (LVEF), i.e. normal filling pressure if E/e’<8 or E/e’ 8–12 and LAVI<34 mL/m2, and high filling pressure if E/e’ 8–12 and LAVI>34 or E/e’>12, respectively [14].

This study was performed in accordance with the Helsinki Declaration and was approved by an institutional review board (the Danish Regional Committee on Biomedical Research Ethics, H-3-2012-005). After receiving oral and written information about the study, all patients gave written informed consent.

### Statistical analyses

All echocardiographic parameters were reviewed for outliers by histogram plots and prespecified cut-offs.

Differences in distribution of CV risk factors, hemodynamic and echocardiographic variables between women with angina and asymptomatic controls were investigated with age-adjusted linear regression analysis for continuous variables and logistic regression for dichotomous variables. Multivariable regression analysis of echocardiographic parameters was used to adjust for age, CV risk factors and medication. A subgroup analysis was performed comparing only patients with either typical angina or dyspnea with controls. Normal distribution was assessed graphically.

Confidence intervals (CI) refer to 95% intervals and a two-sided p-value below 0.05 was considered significant. All analyses were performed using STATA/IC 13.1 (StataCorp LP, College Station, TX, USA).

## Results

Out of 3568 screened women with angina pectoris and no obstructive stenosis at CAG, 2159 met the iPOWER inclusion and exclusion criteria, 963 were reachable and wished to participate and 956 participants (99%) had TTE performed [4]. In total, 214 controls were included. Baseline characteristics for both the patients with angina and the controls are displayed in Table 1. A high prevalence of dyspnea (69%) was observed among patients with angina. The patients with angina were slightly older than the controls (62.1 vs. 60.1 years). After adjustment for age, the patients with angina had a higher burden of CV risk factors with the exception of hypertension: 51% of both patients with angina and controls reported a history of hypertension. Patients with angina had more use of antihypertensive and other CV medication and blood pressure was better controlled, despite the fact that patients with angina had paused their beta-blocker and anti-hypertensive medicine for 24 hours prior to the examination. Heart rate was higher in the patients with angina. After excluding participants receiving prior beta-blocker treatment (n = 291) to avoid influence of withdrawal tachycardia, the difference in heart rate between populations remained statistically significant (p = 0.002). There were 36 patients with permanent or paroxysmal atrial fibrillation. However, only 10 of these patients had atrial fibrillation at time of examination.

**Table 1:** Background characteristics of women with angina and no obstructive coronary artery disease and controls.

	Women with angina (n = 956)	Controls (n = 214)	p-value*
**Descriptive parameters** †			
Age (years), mean (SD)	62.1 (9.7)	60.1 (11.0)	0.008
Body Mass Index (kg/m^2^), mean (SD)	27.2 (5.4)	25.4 (4.4)	<0.001
Hypertension, n (%)	484 (51)	109 (51)	0.35
Diabetes Mellitus, n (%)	126 (13)	5 (2)	<0.001
Smoking (current), n (%)	152 (16)	25 (12)	0.037
Atrial fibrillation, n (%)	36 (4)	0 (0) ll	NR
Dyspnea, n (%)	639 (69)	0 (0) ll	NR
**Hemodynamic parameters** ‡			
Heart rate (beats/min), mean (SD)	69.1 (10.7)	66.6 (9.8)	0.002
Systolic blood pressure (mmHg), mean (SD)	133.0 (21.8)	141.5 (21.7)	<0.001
Diastolic blood pressure (mmHg), mean (SD)	74.3 (19.4)	80.3 (9.8)	<0.001
**Medication,** §			
Acetylsalicylic acid, n (%)	424 (45)	15 (7)	<0.001
Beta-receptor blockers, n (%)	281 (30)	10 (5)	<0.001
Cholesterol lowering drugs, n (%)	475 (50)	30 (15)	<0.001
ACE-inhibitor, n (%)	139 (15)	13 (6)	0.002
Angiotensin-II-receptor blocker, n (%)	175 (19)	11 (5)	<0.001
Calcium antagonist, n (%)	205 (22)	13 (6)	<0.001
Thiazide, n (%)	146 (15)	16 (8)	0.008
Furosemide, n (%)	60 (6)	4 (2)	0.023

*p-value from age adjusted logistic or linear regression analysis. SD: Standard deviation.

†No more than 5 missing values

‡No more than 7 missing values

§No more than 26 missing values.

ll Controls with atrial fibrillation or dyspnea were excluded. NR: not relevant

The patients with angina had greater LV wall dimensions, a higher LVMI and larger LAVI compared with the controls (Table 2). Apart from deceleration time and e’ that were similar across the two groups, indices of LV diastolic function indicated a more impaired LV diastolic function in the patients with angina. E/A-ratio and e’/a’ were reduced in the patients with angina suggesting an impaired myocardial relaxation and reduced LV compliance. E/e’ was increased indicating a higher LV filling pressure. Accordingly, a significantly larger proportion of the patients with angina fulfilled guideline criteria for a high filling pressure (Table 2). For patients with angina, dyspnea was reported in 74% of with high filling pressure compared with 62% in patients with normal filling pressure (p = 0.12).

**Table 2:** Echocardiographic parameters in women with angina and no obstructive coronary artery disease and controls.

	Women with angina (n = 956)	Controls (n = 214)	p-value*
**Left ventricular and atrial dimensions** †			
LVIDd (cm), mean (SD)	4.66 (0.50)	4.58 (0.43)	0.019
LVPWd (cm), mean (SD)	0.83 (0.09)	0.76 (0.08)	<0.001
IVSd (cm), mean (SD)	0.86 (0.09)	0.81 (0.09)	<0.001
LVMI g/m^2^), mean (SD)	71.8 (13.9)	65.7 (12.3)	<0.001
LAVI (ml/m^2^), mean (SD)	29.5 (7.9)	27.0 (5.7)	<0.001
Left ventricular ejection fraction (%), mean (SD)	58.6 (6.0)	57.6 (5.4)	0.026
Relative wall thickness (cm^2^/cm), mean (SD)	0.37 (0.05)	0.35 (0.04)	<0.001
**Left ventricular diastolic function** ‡			
E (cm/s), mean (SD)	73.3 (16.3)	68.4 (14.4)	<0.001
A (cm/s), mean (SD)	73.0 (16.8)	62.2 (17.4)	<0.001
Deceleration time (ms), mean (SD)	183 (32)	179 (30)	0.20
E/A ratio, mean (SD)	1.03 (0.30)	1.18 (0.40)	<0.001
e’(cm/s), mean (SD)	10.0 (2.8)	10.6 (3.3)	0.20
a’ (cm/s), mean (SD)	10.5 (2.6)	9.0 (2.6)	<0.001
E/e’ ratio, mean (SD)	7.9 (2.7)	7.0 (2.2)	<0.001
e’ /a’, mean (SD)	1.03 (0.44)	1.29 (0.61)	<0.001
Estimated high filling pressure ll, n (%)	150 (17)	13 (6)	0.001

*p-value from age adjusted logistic or linear regression analysis. SD: Standard deviation, LVIDd: Left ventricular internal diameter in diastole, LVPWd: Posterior wall thickness in diastole, IVSd: Septum wall thickness in diastole, LVMI: Left ventricular mass index, LAVI: Left atrium volume index.

†No more than 57 missing values

‡No more than 65 missing values

ll Filling pressure was estimated as normal if E/e’ lat<8 or E/e’ lat is between 8–12 and LAVI<34 mL/m2 and high if E/e’ is between 8–12 and LAVI>34 or E/e’>12.

Multivariable regression analyses were performed to examine whether CV risk factors and medication and hemodynamic variables could explain differences between patients with angina and controls in each of the measures of LV diastolic function. The estimated differences between patients with angina and the controls in LV diastolic parameter values remained highly significant after adjustment for heart rate, systolic blood pressure, atrial fibrillation and CV risk factors (Table 3).

**Table 3:** Estimated differences in diastolic and systolic parameters between angina patients and controls from multivariable adjusted regression analyses.

	β (95% CI), adjusted for age only	β (95% CI), multivariable adjusted regression	p-value*
Left ventricular mass index (g/m^2^)	5.73 (3.71; 7.75)	5.54 (3.24; 7.84)	<0.001
Left atrium volume index (mL/m^2^)	2.34 (1.23; 3.45)	1.85 (0.62; 3.07)	0.003
E (cm/s)	5.32 (2.94; 7.70)	3.83 (1.09; 6.57)	0.006
E/A ratio	-0.13 (-0.17; -0.08)	-0.12 (-0.17; -0.07)	<0.001
E/e’	0.68 (0.30; 1.05)	0.50 (0.07; 0.92)	0.02
e’ /a’	-0.22 (-0.28; -0.15)	-0.19 (-0.26; -0.12)	<0.001
	**Odds ratio (CI), adjusted for age only**	**Odds ratio (CI), multivariable adjusted regression**	
High filling pressure ll	2.90 (1.58; 5.34)	2.70 (1.34; 5.47)	0.006

*p-value from separate multivariable logistic or linear regression with each echocardiographic parameters as outcome and a variable indicating population—women with angina/asymptomatic reference population—as an independent variable; adjusted for age, cardiovascular (CV) risk factors, heart rate, systolic and diastolic blood pressure, and CV medication from Table 1 (Echocardiographic parameter = β*”women with angina/controls”+β1*age+β2*”CV risk factors”+β3*”hemodynamic parameters”+β4*”CV medication”). β is the regression coefficient.

Example: The interpretation of the beta-coefficient is that a patient with angina had 5.54 greater LVMI than a control after adjustment for differences between populations. ll Filling pressure was estimated as normal if E/e’<8 or E/e’ is between 8–12 and LAVI<34 mL/m2 and high if E/e’ is between 8–12 and LAVI>34 or E/e’>12. CI: Confidence interval

Across patient groups defined by symptom characteristics as typical angina, atypical angina or non-cardiac chest pain, no differences in echocardiographic parameters were detected. LV filling pressure showed no consistent associations with the burden of angina symptoms. However, patients with high filling pressure reported slightly more physical limitations and lower angina stability (Table 4).

**Table 4:** Burden of angina symptoms distributed between normal and high filling pressure.

	Normal filling pressure (n = 753, 83%)	High filling pressure (n = 150, 17%)	p-value*
**Symptom characteristics for classic chest pain classification**			
Typical angina, n (%)	158 (21)	32 (21)	0.97**
Atypical angina, n (%)	354 (47)	69 (46)	
Non-cardiac chest pain, n (%)	241 (32)	49 (33)	
**Rose’s angina classification**			
Severe definite angina, n (%)	126 (17)	30 (21)	0.34**
Non-severe definite angina, n (%)	195 (27)	31 (22)	
Non-definite angina, n (%)	404 (56)	82 (57)	
**Symptom burden by Seattle Angina Questionnaire** †			
Physical limitation, mean (SD)	75 (23)	69 (22)	**0.05**
Angina stability, mean (SD)	64 (29)	60 (27)	**0.03**
Angina frequency, mean (SD)	76 (23)	74 (23)	0.22
Treatment Satisfaction, mean (SD)	65 (25)	69 (24)	0.40
Perception/Quality of Life, mean (SD)	50 (26)	49 (29)	0.11

*p-value from age-adjusted linear regression.

**p-value from chi-squared analysis.

†Low score indicating higher symptom burden.

Above analyses only including patients with typical angina or patients with dyspnea yielded similar results. Excluding patients with atrial fibrillation (n = 36) from the analysis also yielded similar results.

## Discussion

Higher LV filling pressures and impaired diastolic function were present in more women with angina and no obstructive CAD compared with asymptomatic controls with cardiovascular risk factors.

Women with angina had a higher CV risk factor burden, but the reference population also had risk factors being from a normal background population. Notably, hypertension was equally present in women with angina and controls. The blood pressure level was higher in controls than in women with angina, which could be expected due to more aggressive treatment in women referred for cardiac assessment. When adjusting for differences in CV risk factors, medication and hemodynamic variables between populations, the observed differences in LV diastolic parameter values persisted suggesting that the impaired LV diastolic function in patients with angina could not only be explained by a difference in CV risk factor profile. Differences in echocardiographic parameters between symptomatic patients and the controls are not likely to have clinical implications because the differences are minor and therefore cannot be used in a diagnostic setting. However, this is a group of patients that is at the moment left undiagnosed since we do not know the pathophysiology behind their symptoms. This study showed that these patients had a slightly impaired LV diastolic function compared with a reference population. Typical angina symptoms or burden of symptoms could not indicate which patients had high filling pressure estimated by echocardiography. However, symptoms are very subjective and difficult to assess. The prevalence of typical angina in our population was identical to that of women with obstructive CAD from the CONFIRM registry (coronary CT angiography evaluation for clinical outcomes: an international multicenter registry) [15]. When comparing only patients with typical angina with the controls the same conclusions were reached.

Only a handful of previous small studies have addressed diastolic function in women with angina and no obstructive CAD. The conclusion from these is in concordance with our results supporting that women with angina have a more impaired LV diastolic dysfunction compared with controls [6–8]. It has recently been speculated that CMD through induction of transient myocardial ischemia and fibrosis leads to impairment of LV diastolic function, i.e. that there is a link between angina with no obstructive CAD and heart failure with preserved LVEF (HFpEF) [16]. In fact, prevalence of CMD is high in both patients with HFpEF and patients with angina and no obstructive CAD [4, 5, 17, 18]. In this current population of women with angina we previously found that the prevalence of CMD assessed by transthoracic echocardiography was 26% [4]. Furthermore, many of the women in this study also had dyspnea (~70%). In a recent PET study of patients with angina and no obstructive CAD (n = 201) a link between CMD and diastolic dysfunction was found [19]. However, we have previously reported no association between CMD assessed by TTE and diastolic function (n = 956) in this population [20].

Another possible explanation for the greater LVMI and more diastolic impairment could be that women with no obstructive CAD have a higher degree of myocardial fibrosis due to the higher CV risk factor burden. However, in our study the women with angina and the controls had a similar prevalence of hypertension [21], which is the main contributor to the development of myocardial fibrosis and adverse cardiac remodeling. Furthermore, differences in LV diastolic function were unaffected by adjustment for differences in CV risk factors indicating that the differences in left ventricular diastolic function between angina patients and controls is independent of risk factor burden.

### Strengths and limitations

The Strengths of the study were the size of the study populations and the consecutive recruitment of angina patients making results generalizable to larger clinical populations. The women that were invited and did not wish to participate had a higher prevalence of CV risk factors, which could only underestimate the prevalence of left ventricular diastolic dysfunction [4]. The control group in this study was representative of a background population and thus had a high prevalence of risk factors, including hypertension, making this control group more comparable to the angina patients. This contrasts with other studies that have used healthy women with low risk factor burden as reference [7, 8]. Controls were, however, slightly younger and leaner than the patients with angina, which is why all statistical comparisons were age-adjusted. Echocardiographic parameters were analyzed with a good inter-analyzer reproducibility as documented in a previous publication [9]. The 2009 guideline definition for LV diastolic dysfunction was utilized. The definition was revised in 2016 recommendations. However, because this study was commenced in 2012 not all parameters for diastolic function used in the recommendations for 2016 was measured [14, 22]. Although, many parameters of diastolic function were more impaired in the women with angina there was no difference in e' between groups. Lastly, the present study offered no mechanistic insight and we cannot exclude that the differences between the patients with angina and the controls could in part be due to unmeasured confounding factors such as obstructive sleep apnea, insulin resistance, epicardial fat, and several inflammatory conditions.

## Conclusion

Among women with angina and no obstructive CAD, estimated high filling pressure and parameters of impaired diastolic function were more prevalent except for suggesting a potential link between angina with no obstructive CAD and diastolic dysfunction. However, the common denominators are unknown

## Supporting information

S1 File Data available in STATA format. (DTA)

## Abbreviations

CAD: Coronary artery disease
CAG: Coronary angiography
CV: Cardiovascular
CMD: Coronary microvascular dysfunction
iPOWER: *I*m*p*r*o*ving diagnosis and treatment of *w*omen with angina p*e*ctoris and microvascula*r* disease
LAVI: Left atrium volume index
LV: Left ventricular
LVMI: Left ventricular mass index
TTE: transthoracic echocardiography
